# Polymorphisms in the genes coding for iron binding and transporting proteins are associated with disability, severity, and early progression in multiple sclerosis

**DOI:** 10.1186/1471-2350-13-70

**Published:** 2012-08-10

**Authors:** Donato Gemmati, Giulia Zeri, Elisa Orioli, Francesca E De Gaetano, Fabrizio Salvi, Ilaria Bartolomei, Sandra D’Alfonso, Claudia Dall’Osso, Maurizio A Leone, Ajay V Singh, Rosanna Asselta, Paolo Zamboni

**Affiliations:** 1 Department of Biomedical Sciences & Advanced Therapies, Hematology Unit - Center Hemostasis & Thrombosis, University of Ferrara, Ferrara, Italy; 2 Department of Neurology, Bellaria Hospital, Bologna, Italy; 3 Department of Medical Sciences and IRCAD, Eastern Piedmont University, Novara, Italy; 4 Dipartimento di Biotecnologie Mediche e Medicina Traslazionale, Università degli Studi di Milano, Milan, Italy; 5 Stem Cell and Regenerative Biology (SCRB) Department, Harvard University, Cambridge, MA, 02138, USA; 6 SCDU Neurologia, Ospedale Maggiore della Carità and IRCAD, Novara, Italy; 7 Department of Physics, European School of Molecular Medicine (SEMM), University of Milan, Milan, Italy; 8 Vascular Diseases Center, University of Ferrara, Ferrara, Italy

## Abstract

**Background:**

Iron involvement/imbalance is strongly suspected in multiple sclerosis (MS) etiopathogenesis, but its role is quite debated. Iron deposits encircle the veins in brain MS lesions, increasing local metal concentrations in brain parenchyma as documented by magnetic resonance imaging and histochemical studies. Conversely, systemic iron overload is not always observed. We explored the role of common single nucleotide polymorphisms (SNPs) in the main iron homeostasis genes in MS patients.

**Methods:**

By the pyrosequencing technique, we investigated 414 MS cases [Relapsing-remitting (RR), n=273; Progressive, n=141, of which: Secondary (SP), n=103 and Primary (PP), n=38], and 414 matched healthy controls. Five SNPs in 4 genes were assessed: hemochromatosis (*HFE: C282Y, H63D*), ferroportin (*FPN1: -8CG*), hepcidin (*HEPC: -582AG*), and transferrin (*TF: P570S*).

**Results:**

The *FPN1-8GG* genotype was overrepresented in the whole MS population (OR=4.38; 95%CI, 1.89-10.1; P<0.0001) and a similar risk was found among patients with progressive forms. Conversely, the *HEPC -582GG* genotype was overrepresented only in progressive forms (OR=2.53; 95%CI, 1.34-4.78; P=0.006) so that SP and PP versus RR yielded significant outputs (P=0.009). For almost all SNPs, MS disability score (EDSS), severity score (MSSS), as well as progression index (PI) showed a significant increase when comparing homozygotes versus individuals carrying other genotypes: HEPC -582GG (EDSS, 4.24±2.87 vs 2.78±2.1; P=0.003; MSSS, 5.6±3.06 vs 3.79±2.6; P=0.001); FPN1-8GG (PI, 1.11±2.01 vs 0.6±1.31; P=0.01; MSSS, 5.08±2.98 vs 3.85±2.8; P=0.01); HFE 63DD (PI, 1.63±2.6 vs 0.6±0.86; P=0.009). Finally, HEPC -582G-carriers had a significantly higher chance to switch into the progressive form (HR=3.55; 1.83-6.84; log-rank P=0.00006).

**Conclusions:**

Polymorphisms in the genes coding for iron binding and transporting proteins, in the presence of local iron overload, might be responsible for suboptimal iron handling. This might account for the significant variability peculiar to MS phenotypes, particularly affecting MS risk and progression paving the way for personalized pharmacogenetic applications in the clinical practice.

## Background

Multiple sclerosis (MS) is the leading cause of disability in young and middle-aged people in the developed world. It is an inflammatory, demyelinating disease of the central nervous system (CNS), and is widely considered to have an autoimmune etiology. The multistep mechanism of the disease involves inflammation, demyelination, and neurodegeneration [1].

A growing body of evidence, supported by both post-mortem studies and advanced MRI techniques, shows increased CNS iron stores in MS patients, particularly in the sub-cortical gray matter [2-5]. The hypotheses of iron excess as a cause of oxidative stress [6,7], with possible contribution to neuronal injury and death, has been commonly proposed in other neurodegenerative disorders [5,8,9]. Indeed, iron level manipulation has been reported as being neuroprotective and neurorestorative in neurodegenerative diseases [10]. Moreover, it was demonstrated that iron deficiency provides protection from the development of experimental autoimmune encephalomyelitis, the animal model of MS [11].

A contribution to the development of iron-driven oxidative stress in several degenerative disorders is linked to the presence of one or more genetic variants leading to suboptimal iron balance in the tissue [5,9,12-14]. Some of the main genes and single nucleotide polymorphisms (SNPs) involved in iron management with possible effects on tissue injury are described below.

The *HFE* gene, locus 6p21.3, codes for a membrane protein similar to MHC class I-type proteins. This protein modulates iron absorption by regulating the interaction of the transferrin receptor with transferrin, and defects in this gene cause hereditary hemochromatosis [15]. C282Y and H63D are the two commonest disease-associated variants in the *HFE* gene, and iron-dependent inflammation seems to be influenced by both polymorphisms [12-15]. Apart from hemochromatosis, C282Y increases the risk of iron-dependent skin lesions and affects wound healing in patients with leg iron overload due to chronic venous diseases [14,16]. Among neurodegenerative disorders, the *HFE* gene has been investigated as a modulator of the different clinical phenotypes. In the field of MS, controversial data have been published; C282Y was found to be overrepresented among MS cases of North-Western European origin [17], and it was considered a predictor for early onset, as well as the H63D homozygotes or the H63D/C282Y compound heterozygotes [18]. Other groups did not find any significant association, when comparing MS cases with low versus high disability scores [19]. Nevertheless, the C282Y variant has recently been considered a marker of poorer MS prognosis and it has been associated with MS aggressiveness [20].

The *FPN1* gene, locus 2q32.2, codes for a multiple transmembrane domain protein. Its official name is Solute Carrier Family 40 (iron-regulated transporter), member 1 (SLC40A1). Differently from other iron transporters, it is the only identified mammalian molecule that exports iron outside the cell [21]. *FPN1* expression is finely tuned by the iron responsive element (IRE) in the 5’untranslated region (5’UTR) of mRNA, which, under cell iron overloading, increases protein expression leading to iron exports. Four SNPs and one CGG microsatellite repeat in the *FPN1* gene have been studied in relation to *HFE*[22]. Two of these, -8CG and -98GC, are close to the IRE element and are in complete linkage disequilibrium. To date, no data are available about the role of *FPN1* gene variants in MS susceptibility or in other neurodegenerative disorders, and very few data have been reported on their potential role on other iron overload diseases [16].

The *HEPC* gene, locus 19q13.1, codes for a 25-amino-acid peptide, derived from cleavage of an 84-amino-acid long pro-peptide, which is mainly synthesized by hepatocytes [23]. Its official name is Hepcidin Anti-Microbial Peptide (HAMP), and it is a major regulator of iron balance acting by binding to the FPN1 protein on cell membrane, suppressing it. A polymorphism in the promoter region (−582AG) has recently been described as possibly associated with iron metabolism [24-26], but no data on the *HEPC* gene variants and neurodegenerative diseases are reported so far.

The *TF* gene, locus 3q22.1, codes for a molecule that forms a stable complex with the HFE protein facilitating iron transfer via transferrin receptor [27]. The effect of HFE on iron absorption depends on its relationship with the transferrin receptor: HFE variants affect TF binding, determining a loss of HFE-repressor function for TF uptake, thereby increasing iron transport within the cells. A common variant in the *TF* gene is the P570S (*TF*, C1C2) [28]. The role of the C2 allele in iron balancing [28,29] and in neurodegenerative diseases [30,31] has been debated; nonetheless, a joint effect of the *HFE* and *TF* genes, responsible for a greater synergic effect, suggested possible gene-gene and gene-environment interactions [32].

Considering that little is known and that there are controversial data about the role of iron trafficking genes in the natural history of MS, we decided to investigate whether common functional SNPs within the main iron genes might contribute to MS susceptibility, onset, disability/severity, and progression.

## Methods

### Patients and controls

A total of 414 unrelated patients (female/male = 264/150) affected by clinically definite MS, according to the revised criteria of McDonald [33], and classified according to the criteria of Lublin [34] as having relapsing-remitting (RR, n=273), progressive (n=141), [Secondary (SP), n=103, and Primary (PP), n=38] courses, were enrolled in the study. They were consecutively selected from the patient population of two MS Centres, both placed in Northern Italy (Ferrara/Bologna, n=265; Novara, n=149). Clinical disability and severity were respectively scored using Kurtzke’s Expanded Disability Status Scale (EDSS) and MS Severity Score (MSSS) [35]. The duration of the disease was expressed in years from the date of neurological diagnosis. The progression index (PI), defined as the ratio between EDSS/MS duration, was assessed in the entire MS group. The control group consisted of 414 healthy volunteers matched for age, gender, and geographic origin with the MS patients; control subjects were without any sign or familial history for neurological diseases.

The study was approved by the local Ethical Committee and all the recruited subjects signed an informed consent to participate to the study.

### DNA extraction, PCR conditions, and sequencing

DNA was isolated from peripheral frozen whole blood by the automated DNA extraction and purification robot (BioRobot EZ1 system from QIAGEN; Hilden, Germany), which performs purification of nucleic acids using a magnetic bead technology.

*HFE*, *FPN1*, *HEPC*, and *TF* SNPs were genotyped in the entire case–control cohort by PCR amplifying the relevant genomic region using specific couple of primers and the lyophilic complete UNIVERAL MASTER MIX kit (STAT-NAT DNA-Mix; SENTINEL Diagnostics, Milan, Italy). In all cases, the PCR thermal profile was as follows: 94°/30sec; 57°/30sec; 72°/60sec; x 33 cycles. PCRs were performed in a PTC-200 thermal cycler (M. J. Research, Inc., Watertown, MA, USA). SNPs detection was performed by pyrosequencing using the Pyromark ID System (Biotage AB Uppsala, Sweden) according to the standard procedures for amplicon denaturation, purification, and sequencing. Table 1 shows the primer sequences needed to amplify/sequence the target gene. All the oligo sequences of the SNPs investigated (Forward, Reverse and Sequence primers) were selected to have at least 98.0% compatibility score.

**Table 1:** Primer sequences and restriction-product characteristics

**Oligo name**	**Oligo sequence**	**PCR size (bp)**	**Restriction enzyme**	**Restriction products (bp)**
**FNP1 -8CG**				
**Fw R/P**	5’CCAGTTCCTTGCACTCCTG-3’	129	*BstU*I (60°C)	85+44 (*Pol*)
**Rv R/P**	5’CATCCTCTCTGGCGGTTG-3’ [Bio]			
**Sq**	5’AGAGCCAGCGGGGTC-3’			
**HFE C282Y**				
**Fw R**	5’-TGGCAAGGGTAAACAGATCC-3’	387	*Rsa*I (37°C)	247+140 (*wt*)
**Rv R**	5’-CTCAGGCACTCCTCTCAACC-3’			
**Fw P**	5’-CGAACCTAAAGACGTATTGCC-3’			
**Rv P**	5’-CCCAATAGATTTTCTCAGCTCCT-3’ [Bio]			
**Sq**	5’GGAAGAGCAGAGATATACG-3’			
**HFE H63D**				
**Fw R**	5’-ACATGGTTAAGGCCTGTTGC-3’	207	*Bcl*I (50°C)	137+70 (*Pol*)
**Rv R**	5’-GCCACATCTGGCTTGAAATT-3’			
**Fw P**	5’-CCACATCTGGCTTGAAATTCT-3’			
**Rv P**	5’-GTTTGAAGCTTTGGGCTACG-3’ [Bio]			
**Sq**	5’GGGCTCCACACGGCG-3’			
**TF P570S**				
**Fw R**	5’-GCTGTGCCTTGATGGTACCAGGTAA-3’	110	*BstE*II (60°C)	89+21 (*wt*)
**Rv R**	5’-GGACGCAAGCTTCCTTATCT-3’			
**Fw P**	5’-GAAAAAGACTATGAGTTGCTGTGC-3’			
**Rv P**	5’-CTGTGACCACAGCGTGATTC-3’ [Bio]			
**Sq**	5’-TGATGGTACCAGGAA-3’			
**HEPC -582AG**				
**Fw R**	5’-ACCCTCCTGCCTTGGCCTC-3’	252	*HpyCH4*IV (37°C)	226+26 (*Pol*)
**Rv R**	5’-CCATTGCTTTAAGCTCTCACC-3’			
**Fw P**	5’-ACATCTCAAGGGTCTGACACTGG-3’			
**Rv P**	5’-GAGCAGGGCAAGCATCAGC-3’ [Bio]			
**Sq**	5’-TCTGACACTGGGAAAAC-3’			

Fw and Rv indicate the forward and reverse primer, respectively; Sq indicates the sequencing primer; R and P indicate Restriction and Pyrosequencing technique, respectively; *WT* and *Pol*, indicate the wild-type (common) and polymorphic (rare) allele, respectively; [Bio], indicate the biotinylated primer.

### Genotype confirming procedure

Haplotypes were confirmed by re-genotyping about 20% of randomly selected samples among each different genotype group for each specific polymorphism by means of enzymatic restriction of PCR amplicons. Table 1 shows the restriction enzymes utilized (New England Biolabs Inc., Hitchin, UK), the digestion fragments obtained, and the specific temperature for each different restricted amplicon. All the digestion reactions were carried out according to the Supplier’s instructions. There were no discrepancies between genotypes determined in duplicate and/or by different methods. Known genotypes were used as control references.

### Statistical analysis

Statistical differences among groups were assessed by the Student’s *t*-test and the Chi-squared test, respectively, for mean values and genotype distribution comparisons. When appropriate, Yates' correction or Fisher's exact test was applied. Adjusted Odds Ratios (OR) and 95% confidence intervals (95%CI), calculated by logistic regression models, were used to estimate the risk associated with MS and to the different subtypes in the presence of the rare homozygous condition (e.g. *FPN1* -8GG, *HFE* 63DD, *HEPC* -582GG, and *TF* 570SS) or heterozygous (*HFE* 282CY) condition compared to the remaining genotypes (i.e. heterozygous and/or homozygous for the common allele). The model accounted for sex and age distribution between cases and healthy controls. P values are presented both as uncorrected (if ≤ 0.05) and as corrected for multiple testing (Bonferroni correction).

Power estimates indicated that, if each analyzed polymorphism (disease allele frequency of 10%) was to directly confer a 1.5 to 2-fold increase in the relative risk of MS, the case/control cohort used in this research would be of sufficient size to have 76 to 100% power to detect a significant association at the 0.05 level (the power decreases to 54 and 99% for α=0.01).

Survival curves were constructed by the Kaplan-Meier method, and survival among groups was compared using the Log-Rank test and the associated risk values were examined using a Cox-proportional hazard model. The end-point was the date of starting progression or the tenth year of follow-up, whichever came first.

All analyses were performed by using Systat V.5.0 (Systat Inc., Evanston, IL, USA) and the SPSS Statistical Package (SPSS Inc., Chicago, IL, USA).

## Results

### Population characteristics

The clinical and demographic characteristics in the whole MS group, in the MS subgroups, and in healthy controls are shown in Table 2. As expected, progressive course had significantly higher EDSS, PI, and MSSS when compared to the RR subgroup. Progressive cases had significant longer disease duration and higher mean age at recruitment. Accordingly, they showed the highest PI value, whilst the other clinical findings were not significantly different among subgroups.

**Table 2:** Patients’ and healthy controls’ characteristics

	**Whole group**	**RR**	**PP + SP**	**Healthy controls**
	**(n=414)**	**(n=273)**	**(n=141)**	**(n=414)**
**female/male**	264/150	179/94	85/56	264/150
**♀ (%)**	(63.77 %)	(65.56%)	(60.3%)	(63.77 %)
**Age, yy ± SD**	42.0±11.0	38.5±9.51	48.5±10.40*	42.0±11.0
**(range)**	(16.0-72.0)	(17.0-70.0)	(16.0-72.0)	(16.0-72.0)
**onset, yy ± SD**	32.37±10.37	31.65±10.31	33.17±10.21	-- --
**(range)**	(11.0-61.0)	(14.0-61.0)	(11.0-55.0)	
**Duration, yy ± SD**	9.14±7.43	6.92±6.47	13.44±7.34*	-- --
**(range)**	(0.2-50.0)	(0.2-50.0)	(0.2-35.0)	
**EDSS ± SD**	2.91±2.24	1.8±1.04	5.03±2.43*	-- --
**(range)**	(1.0-10.0)	(1.0-7.0)	(1.0-10.0)	
**PI ± SD**	0.63±1.08	0.59±0.94	0.72±1.32*	-- --
**(range)**	(0.03-10.0)	(0.03-10.0)	(0.04-10.0)	
**MSSS ± SD**	3.94±2.74	2.93±1.99	5.98±2.87*	-- --
**(range)**	(0.13-9.99)	(0.13-9.6)	(0.29-9.99)	

^*^P<0.0001, when compared to the RR group. Values shown are mean, standard deviation (SD), and (ranges).

### SNP genotypes and MS susceptibility

All the investigated SNP genotypes were distributed according to the Hardy-Weinberg equilibrium in both case and healthy control groups.

Table 3 shows the genotype distributions and the associated ORs computed in the total MS patients and in the clinical subtypes compared to healthy controls or, when specified, to the RR subgroup.

**Table 3:** Genotype distributions and related OR values

	***FPN1*-8CG**			***HFE*H63D**			***HFE*C282Y**		***HEPC*-582AG**			***TF*P570S**		
**Genotypes (%)**	**CC**	**CG**	**GG**	**HH**	**HD**	**DD**	**CC**	**CY**	**AA**	**AG**	**GG**	**PP**	**PS**	**SS**
**All cases (n=414)**	244 (58.9)	141 (34.05)	29 (7.0)	288 (69.6)	113 (27.3)	13 (3.15)	401 (96.9)	13 (3.1)	205 (49.5)	175 (42.27)	34 (8.2)	278 (67.15)	122 (29.5)	14 (3.4)
**OR (95%CI)**	4.38 (1.89-10.1)			1.65 (0.67-4.01)			0.76 (0.36-1.58)		1.45 (0.85-2.5)			1.3 (0.6-2.86)		
**P uncorrected**	P<0.0001			(NS)			(NS)		(NS)			(NS)		
**(P corrected)**	(P<0.0004)													
**RR (n=273)**	162 (59.3)	92 (33.7)	19 (7.0)	190 (69.6)	77 (28.2)	6 (2.2)	266 (97.4)	7 (2.6)	144 (52.75)	114 (42.0)	15 (5.5)	189 (69.2)	75 (27.5)	9 (3.3)
**OR (95%CI)**	4.35 (1.8-10.5)			1.1 (0.4-3.32)			0.61 (0.25-1.5)		0.94 (0.49-1.83)			1.25 (0.51-3.05)		
**P uncorrected**	P<0.0001			(NS)			(NS)		(NS)			(NS)		
**(P corrected)**	(P<0.0004)													
**PP + SP (n=141)**	82 (58.2)	49 (34.7)	10 (7.14)	98 (69.5)	36 (25.5)	7 (4.9)	135 (95.7)	6 (4.3)	61 (43.3)	61 (43.3)	19 (13.5)	89 (63.1)	47 (33.3)	5 (3.5)
**OR (95%CI)**	4.21 (1.57-11.28)			2.65 (0.94-7.45)			1.04 (0.4-2.69)		2.53 (1.34-4.78)			1.35 (0.46-3.95)		
**P uncorrected**	P=0.003			(NS)			(NS)		P=0.006			(NS)		
**(P corrected)**	(P=0.012)								(P=0.024)					
**OR1 (95%CI)**	1.02 (0.46-2.26)			2.32 (0.77-7.05)			1.69 (0.56-5.12)		2.68 (1.32-5.45)			1.08 (0.35-3.28)		
**P uncorrected**	(NS)			(NS)			(NS)		P=0.009			(NS)		
**(P corrected)**									(P=0.036)					
**Controls (n=414)**	278 (67.1)	129 (31.2)	7 (1.7)	305 (73.7)	101 (24.4)	8 (1.9)	397 (95.9)	17 (4.1)	238 (57.5)	152 (36.7)	24 (5.8)	280 (67.6)	123 (29.7)	11 (2.7)

All OR calculations are obtained computing the rare homozygous genotype vs the rest of genotypes comparing cases vs controls. OR_1_ is referred to the Progressive group in which the reference category is the RR subgroup. Corrected and uncorrected P-values are respectively referred to the presence/absence of Bonferroni correction. NS, not significant.

Globally, the rate of *FPN1* -8GG homozygotes was 7.0% in MS cases and 1.7% in controls. This yielded an overall OR of 4.38 (95%CI, 1.89-10.1; P<0.0001) when compared with the rest of genotypes. Among RR and Progressive cases computed together (SP + PP), the assessed risks were similar to that of the entire MS population (OR=4.35; 95%CI, 1.8-10.5; P<0.0001, and OR=4.21; 95%CI, 1.57-11.28; P=0.003, respectively). Finally, no comparisons showed a significant difference in genotype distribution between RR and Progressive cases.

As far as *HFE* gene polymorphisms are concerned, H63D yielded ORs>1 in all the considered subgroups, though far from statistical significance. C282Y yielded non-significant ORs≤1 in many of the considered subgroups. Significant ORs were not found in combined analyses computing C282Y/H63D double carriers, neither in the whole nor in the subgroups (data not shown).

Considering the *HEPC* -582AG variant, significant risk values were restricted to the Progressive group, when compared either with healthy controls (OR=2.53; 95%CI, 1.34-4.78; P=0.006) or RR cases (OR=2.68; 95%CI, 1.32-5.45; P=0.009). Although we do not show data in detail, we evidenced that the risk further increased among PP patients with values higher than 4-fold (OR=4.4; 95%CI, 1.83-10.5; P=0.001). Due to the scanty number of PP cases in our study, all the related results could be featured by chance, nevertheless, it is noteworthy a clear stepwise trend of GG homozygote frequency from RR (5.5%), to SP (10.7%), to PP (21.1%). This yielded a significant over-representation of GG homozygotes among the whole Progressive group (13.5%) when compared to controls (5.8%; P=0.006) or RR sub-group (5.5%; P=0.009). It could be speculated that MS patients carrying the G-allele might be at increased risk for progression.

No risk association was found considering the *TF* P570S gene variant, in the whole as well as in the different subgroups considered, though appreciable ORs>1 were found.

The Bonferroni correction, applied to the genotype comparison, confirmed all the significances obtained in the uncorrected analysis (Table 3).

When allelic comparisons were performed, the significant overrepresentation of the rare allele in patients was completely retained for each SNP investigated and in every group/subgroup resembling those of the genotype. However, after Bonferroni correction the number of significances was cut down (Table 4).

**Table 4:** Allelic distributions and related OR values

	***FPN1-*8CG**		***HFE*H63D**		***HFE*C282Y**		***HEPC-*582AG**		***TF*P570S**	
**Allele (%)**	**C**	**G**	**H**	**D**	**C**	**Y**	**A**	**G**	**P**	**S**
**All subjects (n=828)**	629 (76.0)	199 (24.0)	689 (83.2)	139 (16.8)	802 (96.86)	26 (3.14)	585 (70.6)	243 (29.3)	678 (81.9)	150 (18.1)
**OR (95%CI)**	1.52 (1.2-1.93)		1.23 (0.94-1.6)		0.76 (0.45-1.27)		1.30 (1.05-1.62)		1.04 (0.81-1.34)	
**P uncorrected**	<0.0001		NS		NS		0.020		NS	
**(P corrected)**	(<0.002)		(NS)		(NS)		(NS)		(NS)	
**RR (n=546)**	416 (76.2)	130 (23.8)	457 (83.7)	89 (16.3)	532 (97.4)	14 (2.6)	402 (73.6)	144 (26.4)	453 (83.0)	93 (17.0)
**OR (95%CI)**	1.5 (1.15-1.95)		1.18 (0.88-1.6)		0.61 (0.33-1.16)		1.12 (0.88-1.44)		0.97 (0.73-1.29)	
**P uncorrected**	0.004		NS		NS		NS		NS	
**(P corrected)**	(NS)		(NS)		(NS)			(NS)		(NS)	
**PP + SP (n=282)**	213(75.5)	69 (24.5)	232 (82.3)	50 (17.7)	270 (96.86)	12 (3.14)	183 (64.9)	99 (35.1)	225 (79.8)	57 (20.2)	
**OR (95%CI)**	1.55 (1.12-2.15)		1.31 (0.91-1.88)		1.04 (0.53-2.03)		1.7 (1.27-2.27)		1.19 (0.85-1.68)		
**P uncorrected**	0.010		NS		NS		<0.0001		NS		
**(P corrected)**	(NS)		(NS)		(NS)		(<0.002)		(NS)		
**OR**_**1**_**(95%CI)**	1.03 (0.74-1.45)		1.10 (0.75-1.61)		1.7 (0.8-3.7)		1.51 (1.11-2.06)		1.23 (0.86-1.78)		
**P uncorrected**	NS		NS		NS		0.010		NS		
**(P corrected)**	(NS)		(NS)		(NS)		(NS)		(NS)		
**Controls (n=828)**	685 (82.7)	143 (17.3)	711 (85.9)	117 (14.1)	794 (95.9)	34 (4.1)	628 (75.9)	200 (24.1)	683 (82.5)	145 (17.5)	

All OR calculations are obtained computing the rare vs the common allele comparing cases vs controls. OR_1_ is referred to the Progressive group in which the reference category is the RR subgroup. Corrected and uncorrected P-values are respectively referred to the presence/absence of Bonferroni correction. NS, not significant.

### SNPs genotypes and MS clinical characteristics (single analyses)

Table 5 shows the clinical characteristics (age of onset, disease duration, EDSS, PI, and MSSS) in the whole group of MS patients stratified by the different SNP genotypes.

**Table 5:** Clinical findings stratified by SNPs in the whole group of patients

**FPN1 -8CG**	**Onset**	**Duration**	**EDSS**	**PI**	**MSSS**
**−8CC**	33.5±9.8	9.3±7.35	2.73±2.07	0.58±0.78	3.72±2.66
**(n=244)**	(11.0-60.0)	(0.3-38.0)	(1.0-10)	(0.03-7.0)	(0.13-9.99)
**−8CG**	31.92±10.45	9.32±7.95	3.07±2.48	0.64±1.24	4.09±2.78
**(n=141)**	(14.0-61.0)	(0.2-50.0)	(1.0-9.0)	(0.03-10)	(0.15-9.97)
**−8GG**	33.38±12.3	7.0±5.17	3.59±2.43	1.11±2.01	5.08±2.98
**(n=29)**	(15.0-53.0)	(0.2-20.4)	(1.0-9.0)	(0.1-10.0)	(0.78-9.97)
**P uncorrected**	NS	0.05	0.045	0.01	0.01
**(P corrected)**				(0.05)	(0.05)
***HFE C282Y***					
**282CC**	32.3±10.37	9.19±7.49	2.9±2.25	0.63±1.07	3.9±2.73
**(n=401)**	(11.0-60.0)	(0.2-50.0)	(1.0-10)	(0.03-10.0)	(0.13-9.99)
**282CY**	33.62±11.12	7.27±5.35	3.16±2.21	0.58±1.54	4.64±2.91
**(n=13)**	(16.0-61.0)	(1.0-27.0)	(1.0-9.0)	(0.13-1.3)	(1.13-9.92)
***P-value***	NS	NS	NS	NS	NS
***HFE H63D***					
**63HH**	32.75±10.94	8.92±7.12	2.89±2.23	0.62±0.98	3.99±2.78
**(n=288)**	(11.0-61.0)	(0.2-50.0)	(1.0-10)	(0.03-10)	(0.15-9.99)
**63HD**	30.97±8.94	9.8±8.2	2.81±2.21	0.55±1.01	3.65±2.55
**(n=113)**	(14.0-56.0)	(0.5-38.0)	(1.0-9.0)	(0.03-10)	(0.13-9.97)
**63DD**	36.1±8.23	8.43±7.57	3.96±2.93	1.63±2.6	5.33±3.03
**(n=13)**	(28.0-55.0)	(0.2-22.2)	(1.0-8.5)	(0.2-7.5)	(1.28-9.99)
**P uncorrected**	0.06	NS	NS	0.009	0.03
**(P corrected)**	(NS)			(0.045)	(NS)
***HEPC -582AG***					
**−582AA**	29.57±9.86	9.54-7.3	2.59±2.06	0.57±.1.14	3.39±2.56
**(n=205)**	(14.0-61.0)	(0.2-38.0)	(1.0-9.0)	(0.03-10)	(0.13-9.97)
**−582AG**	35.1±9.92	8.78±7.78	3.01±2.22	0.63±0.94	4.26±2.7
**(n=175)**	(11.0-56.0)	(0.2-50.0)	(1.0-9.0)	(0.04-10.0)	(0.29-9.98)
**−582GG**	35.2±11.56	8.55±6.45	4.24±2.87	0.96±1.38	5.6±3.06
**(n=34)**	(20.0-56.0)	(0.5-27.0)	(1.0-10)	(0.13-7.0)	(1.13-9.99)
**P uncorrected**	0.07*	NS	0.003	0.08	0.001
**(P corrected)**	(NS)		(0.015)	(NS)	(0.005)
***TF P570S***					
**570PP**	32.38±10.44	8.42±6.97	2.72±2.15	0.69±1.26	3.78±2.66
**(n=278)**	(11.0-56.0)	(0.2 34.0)	(1.0-10)	(0.03-10.0)	(0.15-9.99)
**570PS**	32.32±10.53	11.08±8.24	3.38±2.41	0.48±0.52	4.28±2.92
**(n=122)**	(14.0-61.0)	(0.5-50.0)	(1.0-9.0)	(0.03-4.0)	(0.13-9.97)
**570SS**	32.53±8.43	6.52±6.17	2.47±2.05	0.77±0.89	4.1±2.52
**(n=14)**	(21.0-51.0)	(0.5-22.0)	(1.0-9.0)	(0.15-3.0)	(1.13-9.73)
**P uncorrected**	NS	0.06	NS	NS	NS
**(P corrected)**		(NS)			

Values shown are mean, standard deviation (SD), and (ranges). All P-values shown are obtained computing the rare homozygous genotype vs the rest of genotypes. *Computing G-carriers vs AA-genotype P-value <0.0001. Significant P-values, and those <0.10 are reported. NS, not significant.

*FPN1* -8GG homozygotes had a slightly higher mean EDSS score when compared with the rest of genotypes (3.59±2.43 vs 2.85±2.45; P=0.045). Although a trend among the three different genotypes was observed, it did not reach significance (P-trend= 0.07). The same EDSS comparisons yielded indeed higher significant differences in the RR subgroup (2.59±2.12 vs 1.74±0.88; P=0.0006; P-trend= 0.01). Similarly, considering PI, *FPN1* -8GG homozygotes had a significantly higher index when compared with the remaining genotypes (1.11±2.01 vs 0.59±0.97; P=0.01) and the significant trend among genotypes was maintained, as well as PI comparisons among the RR subgroup (1.08±2.2 vs 0.55±0.76; P=0.01; P-trend=0.03). MSSS significantly rose among the -8GG homozygotes in the whole (5.08±2.98 vs 3.85±2.7; P=0.01), as well as in the RR subgroup (4.02±2.99 vs 2.85±1.87; P=0.01).

*HFE* polymorphisms showed PI and MSSS values significantly related to the H63D gene variant exclusively in the whole group. Accordingly, by comparing 63DD homozygotes with the remaining cases, PI was significantly higher (1.63±2.6 vs 0.59±0.99; P=0.009) as well as MSSS did (5.33±3.03 vs 3.89±2.72; P=0.03). Concerning the *HFE* C282Y polymorphism, none of the clinical characteristics were significantly related with particular genotypes. This was very likely due to the rarity of 282Y carriers (e.g. no 282YY homozygotes, were found).

The *HEPC* -582AG variant had a higher mean EDSS value among -582GG homozygotes compared with the other genotypes (4.24±2.87 vs 2.78±2.18 P=0.003). Similarly, MSSS showed higher values among GG-homozygotes (5.6±3.06 vs 3.79±2.65; P=0.001). Conversely, PI values did not reach significant changes (P=0.08), as well as further sub-analyses.

*TF* P570S in our study population did not affect at significant extent any clinical finding, neither in the whole, nor in the subgroups.

Interestingly, an unexpected, significant delay in onset (about 6-yy) was observed among *HEPC −*582 G-carriers respect to non carriers (35.11±10.3 vs 29.57±9.86; P<0.0001). A similar behaviour, though at a lesser extent, and restricted just to homozygotes, was observed among the *HFE* H63D variant (36.1±8.23 vs 32.24±10.11; P=0.06).

Finally, disease duration did not show significant differences either in the whole or in the subset groups.

### SNPs genotype, MS susceptibility and clinical characteristics (combined case–control analysis)

In attempt to calculate a cumulative MS risk associated with the coexistence of multiple predisposing genotypes, we compared the whole group of cases and controls carrying a combination of at least four risk alleles in at least two different SNPs (multi-carriers) with subjects who were homozygous for the common allele in all the considered gene variants (fully wild-types). Combined homozygotes at least in two different SNPs, single homozygotes in one and combined carriers in at least two, or carrying at least a quadruple heterozygous condition, they globally were 12.1% in patients (n=50) and 5.1% in controls (n=21). Conversely, the fully wild-type condition was 11.4% in cases (n=47) and 17.9% in controls (n=74). Strongly significant risk-values were obtained from this kind of comparison, suggesting a hypothetical cumulative risk measurability (OR=3.74; CI95%, 2.0-7.02; P<0.0001), although no synergistic effects were recorded.

### Combined intra-case analysis

Similarly, to verify the effects of the combined carrier condition on MS, we stratified all the clinical characteristics investigated by multi-carrier genotype conditions. We found that the combined carrier patients had higher mean values of EDSS (3.65±2.71 vs 2.07±1.5; P=0.0007), PI (1.0±1.4 vs 0.35±0.45; P=0.006), and MSSS (5.06±2.9 vs 2.7±2.12; P=0.0007) when compared with the fully wild-type patients (Table 6). Accordingly, in the combined carriers mean EDSS increased about 1.8-fold, mean PI 2.86-fold, and mean MSSS 1.9-fold.

**Table 6:** Clinical finding comparisons between multi-carriers and fully wild-types

	**Onset**	**Duration**	**EDSS**	**PI**	**MSSS**
**Multi-carriers (n=50)**	36.23±9.09 (20–53)	7.83±6.42 (1.0-22)	3.65±2.71 (1.0-9.0)	1.0±1.4 (0.09-4.0)	5.06±2.9 (0.85-9.97)
**Fully wild-types (n=47)**	33.26±10.39 (16–55)	8.78±5.57 (0.5-24)	2.07±1.5 (1.0-7.5)	0.35±0.45 (0.07-2.24)	2.7±2.12 (0.45.-8.64)
**P uncorrected**	NS	NS	0.0007	0.006	0.0007
**(P corrected)**			(0.0035)	(0.03)	(0.0035)

Multi-carriers (patients carrying at least four rare alleles in at least two different genes) and fully wild-types (homozygous patients for the common allele in all the considered genes) are as defined in the Results section. Values shown are mean, standard deviation (SD), and (ranges). NS, not significant.

### Retrospective survival analysis among SP and RR patients

**Figure 1:**
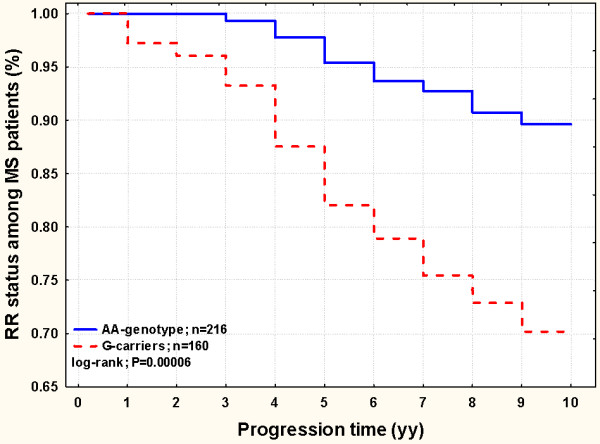
**Survival analysis among 376 MS patients (SP + RR; n=103+273) stratified by the* HEPC *-582AG SNP.** The survival trend of the RR status among MS patients was significantly different when stratified by HEPC SNP. The comparison yields an increased chance to progress in the secondary progressive MS course among G-carriers (dashed line) (HR=3.55; 1.83-6.84; log-rank P=0.00006).

In order to verify the hypothesis that MS patients carrying the *HEPC* -582G-allele might be at increased risk for progression, we calculated among the 103 SP patients, how long they stayed within the previous and less severe clinical phenotype (i.e. the RR condition) before they switched towards the severest SP condition, and this was stratified by the SNPs investigated. *HEPC* -582AG showed an extraordinary output, ascribing to the G-allele the role of earlier *progression-switch*. In detail, after a retrospective observational analysis of ten years, patients carrying the -582G-allele had a higher chance to progress into the SP-phenotype of almost 3-fold (HR=2.77; 1.45-5.34; log-rank P=0.001) if compared to patients carrying the -582AA counterpart genotype. This partial observation prompted us to also include in the survival analysis all the RR patients (n=273), totally analyzing 376 MS patients (Figure 1). The overall HR was greatly improved (HR=3.55; 1.83-6.84; log-rank P=0.00006). Among the other analyzed SNPs, no similar results or combined effects were observed.

**Figure 2:**
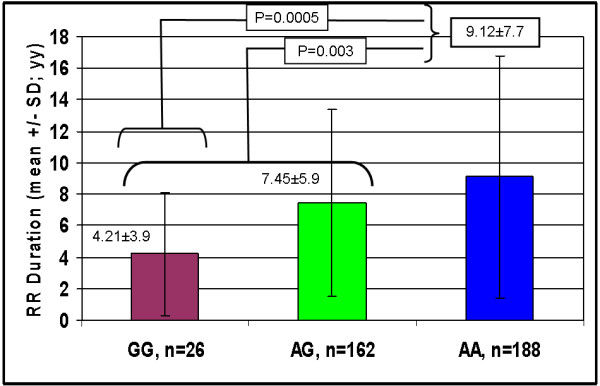
**Mean ± SD (standard deviation) disease duration (years) among 376 MS patients (SP + RR; n=103+273) stratified by the* HEPC *SNP.** The comparison between the *HEPC* genotype conditions yields significant differences (i.e. G-carriers have shorter mean duration time).

An additional indirect result in favour of this hypothesis was obtained by comparing the RR mean disease duration among the three different genotypic classes. Again, -582GG patients showed the shortest disease duration. In detail, the mean duration time decreased as the number of the -582G allele increased (RR, n=273) (GG, 4.52y±3.6 < AG, 6.2y±5.6 < AA, 7.8y±6.8; P=0.007). Similarly to the previous survival analysis, we also included the RR durations of the SP patients (RR + SP; n=376). Accordingly, the significance strongly increased (GG, 4.21y±3.9 < AG, 7.45y±5.9 < AA, 9.12y±7.7; P=0.0005; Figure 2).

### Gender-related sub-analyses

In order to check any gender-related association between MS and the SNPs, we contextually analyzed clinical findings and susceptibilities by gender in every SNP investigated. The main noteworthy combinations are reported below.

Among SP male patients, a 9-fold MS susceptibility increase (OR=8.56; 95%CI, 2.03-36.1; P=0.003) was associated with FPN1 -8GG genotype.

Among Progressive female patients computed together (PP + SP), a high MS susceptibility increase (OR=6.02; 95%CI, 1.1-33.49; P=0.04) was associated with the HFE 63DD genotype. It is to note that females had higher risk also in the whole MS group (OR=3.81; 95%CI, 0.95-20.01; P=0.05).

Finally, among Progressive male patients computed together (PP + SP), a 5-fold MS susceptibility increase (OR=4.9; 95%CI, 1.9-12.5; P=0.001) was associated with the HEPC -582GG genotype.

## Discussion

Several issues surround iron and neurodegenerative disease, due to the fact that iron is essential in neuronal cell life, yet brain iron accumulation can be toxic [5,7-9].

Iron imbalance is strongly suspected in MS pathogenesis, even though there is no evidence that systemic iron overload occurs more frequently in MS patients than in general population [36,37].

In contrast, at the brain level, susceptibility weighted imaging MRI techniques permit to reliably measure iron in the brain and to follow the natural history of iron accumulation. Interestingly, a correlation exists between iron storages and disability, manifested either by cognitive or motor symptoms, suggesting a role in the complex mosaic of MS pathogenesis [38-41].

The exact underlying mechanism by which brain iron accumulates in CNS of MS patients is not fully understood. Iron enters into the brain through the blood–brain-barrier, due to iron transport proteins expressed locally [42] and it is stored according to the efficiency of the transferring receptors. This can be controlled at the post-transcriptional level by iron regulatory proteins (IRPs) that interact with IRE motifs on mRNA to alter the expression on brain endothelial cells, neurons, glia, oligodendrocytes, and macrophages [43,44]. When there is not enough iron in the milieu, IRPs bind IRE motifs to contextually decrease the expression of ferritin and ferroportin and increase that of the transferrin receptor, favouring mRNA stability. Basically, this allows the cell to uptake more iron to efficiently use it before it bounds to the storage protein ferritin [43]. In the literature there are two main hypotheses on the mechanism leading to iron accumulation in brain parenchyma in course of MS. The first is linked with microglia and astrocyte iron accumulation in course of unknown steps linked with neurodegeneration [5,9,44]. The second is linked with a vascular condition, known as chronic cerebrospinal venous insufficiency (CCSVI) related to reduced brain perfusion [45]. It has been hypothesized that CCSVI might favourite erythrocytes diapedesis, and subsequent iron deposition [12,13,38,46]. Even though this is an intriguing and interesting hypothesis and a genetic dependence of CCSVI has recently been described by our group [47], other authors do not directly link CCSVI with increased iron and MS [48-50].

Therefore, in spite of the lack of concordance between blood and brain iron levels, whatever the mechanism causing brain iron deposition is, the same group of proteins regulate iron influx, efflux and storage [42,51]. We hence looked at the commonest SNPs in the main iron-protein genes.

The main finding of our study was an increased MS susceptibility risk, of more than 4-fold, associated with the *FPN1* -8GG homozygous genotype. In addition, stratifying disease progression and severity by *FPN1* genotypes, PI and MSSS gradually increased as the number of the G alleles increased, ascribing to the GG-genotype the highest value. This suggests that MS patients carrying the -8G-allele might be at increased risk for disease worsening. These results can really be considered novel and peculiar findings in the field of MS since, to date, *FPN1* SNPs have been only associated with particular diseases, such as venous leg ulcers [16], or reinvestigated as genetic modifiers of *HFE*[22]. *FPN1* expression is regulated at different levels: by the IRE sequence in the 5’-UTR that, interacting with the IRPs, finely tunes how many FPN1 molecules can be expressed [43]; and post-translationally by the hepatic hormone hepcidin [23]. The IRE region, results in increased/reduced *FPN1* expression respectively under high/low cellular iron, leading to personalized iron export. Hepcidin interacts and blocks FPN1 in the presence of high iron levels. Generally, *FPN1* mutations return a molecule that cannot reach the cell surface or block FPN1 internalization and degradation affecting both hepcidin interaction and iron export. The strong closeness of *FPN1* -8CG to the crucial IRE region, prompted us to investigate its role in MS. The significant associations we found can be speculatively interpreted as a direct role on the IRE-IRP interactions, or as an indirect role of still unknown molecular defects in linkage with the SNP. In CNS cells, or in macrophages, these situations may potentially affect iron-balancing, similarly as described for the *HFE* C282Y [52]. Micro-deletions in the IRE region lead to expected increased in *FPN1* levels despite low cellular iron levels, and to date no mutations specifically affecting IRE have been identified in the *FPN1* gene [53].

Our second relevant finding was related to the *HEPC* gene. Homozygous -582GG cases had an increased MS susceptibility of about 2.5-fold among progressive patients and the risk was kept when progressive cases were compared to the RR course. In addition, EDSS progressively increased among the three different *HEPC* genotypes, with homozygotes about 1.5-higher than the rest of cases. Noteworthy, the rate of -582GG homozygotes was higher among progressive cases (13.5%) when compared to RR group (5.5%), who retained the same rate observed among healthy controls (5.8%). This could suggest that those patients might have rapid disease progression and/or higher chance for progression. Though confounding, due to the unavoidable presence of a great proportion of RR who will develop secondarily the progressive clinical course, this result could even be underestimated, because of the few homozygotes found among RR could even decrease after progression, and improve the statistical comparison. To verify the hypothesis, we split the group of SP cases (n=103) in those with/without the -582G-allele in order to calculate how long these two subgroups stayed in the previous less severe clinical phenotype before becoming progressive. Indeed, during a ten-year retrospective analysis, those carriers had a 3-fold higher chance to progress in the SP-phenotype if compared to the counterpart -582AA genotype. Similarly, including in the same survival analyses also the RR patients, those carriers had a 4-fold higher chance to progress. If this was true, the complementary analysis, that is computing together the mean duration time of the RR-patients (n=273) and that of the previous RR status of the 103 SP patients (total, n=376), could indirectly confirm this hypothesis by yielding opposite results (i.e. -582AA-carriers show a longer disease duration). That is exactly what we observed (GG, 4.52y±3.6 < AG, 6.2y±5.6 < AA, 7.8y±6.8); a possible explanation is that SP G-carriers could have faster left the RR condition to switch in the progressive form. Therefore, the RR G-carriers could have a potential shorter mean duration time within the less severe condition (GG, 4.21y±3.9 < AG, 7.45y±5.9 < AA, 9.12y±7.7). We recognize the intrinsic limit of these partial and indirect results, but all are in favour of an *earlierprogression-switch* role ascribable to the *HEPC* polymorphism. Conflicting and scanty results exist on the -582AG *HEPC* variant [24,25]. The G-allele decreases the transcriptional activity by 20% respect to the A-allele in HepG2 cells in the presence of upstream stimulatory factor 1 (USF1) and by 12-14% with USF2 [26]. The Authors concluded that the promoter variant is not associated with serum iron parameters and that the *in-vitro* studies resulted in little reductions of the G-allele mediated trans-activation. Although they ascribed to the *HEPC* variant negligible *in-vivo* effects, we state that, regardless the small change in the promoter activity between the two alleles, this could be enough to have significant detrimental effects on long-staying iron overload as is the case in MS patients. Accordingly, also subtle chronic lower HEPC expressions in subjects with -582G-allele may be responsible for significant local iron dysregulation mostly in homozygous GG-patients. We previously reported that even minor SNP effects (i.e. those found in MMP12 -82AG) had significant results in another degenerative disease under chronic iron-overload conditions [16].

As far as the *HFE* gene is concerned, H63D and C282Y did not reveal in our population associations with MS. One exception was the 3-fold higher PI found among the 63DD-homozygotes. However, also in other studies the role of the *HFE* gene in MS, seems not to be particularly decisive, being often controversial [17-20]. HLA-DR15 is associated with younger age of onset in MS [54], though we found an appreciable delayed onset among HFE63 DD-Homozygotes. This could be explained by speculating that iron greedy-cells (i.e. those with the polymorphism) could even be protective, paradoxically helping myelin synthesis in the early phases of the disease [55]. After iron moves on insoluble-hemosiderin, iron-starved cells cannot use it, this favours energy crisis and cell apoptosis [5,9,56]. Similarly, this could also be speculated for the *HEPC* variant, in which G-carriers show delayed onset.

Controversial results exist in the association between *TF* P570S and Alzheimer disease (AD) [30,31], hypothesizing a not definitively demonstrated defect in total iron binding capacity [28,29] and a suggestive synergism between *TF* and *HFE* gene variants and AD [32]. We did not find such a synergism in MS, except a non-significant higher MSSS among the *TF* 570S-carriers.

Gender appears to play critical role in development, progression and treatment of MS. In addition, higher brain iron level was found associated with male gender in presence of common iron gene SNPs [57,58]. For this reason, we performed a gender-related sub-analysis, and we found different risk associations related to the different SNPs considered, but definite results cannot be drawn due to the low number of patients obtained after subanalyses. Clarifying a possible differential gender-associated risk to develop neurodegenerative diseases, combining genetic and MRI biomarkers, may help clinicians to design primary intervention programs to select high-risk sub-groups.

We conclude that, all the SNPs investigated work in the same direction: potential iron dysregulation, oxidative tissue damage, and possible actions on MS [51]. This was the reason we looked at the combined effect that the coexistence of several at risk-alleles might have on MS. The fact that among multi-carriers the risk increased, as well as disability, progression, and severity did, strongly implies the multi-gene nature of iron unbalancing in MS.

We recognize that the main limitation of our study is linked to the low number of investigated SNPs. A relevant number of SNPs exist in other candidate genes related to tissue inflammation and degeneration. A further shortcoming in the interpretation of our results is linked with the lack of knowledge still present in MS pathogenesis as well as in the steps leading to iron accumulation.

## Conclusions

Whatever the mechanism causing brain iron deposition is, our study shows strong influence of gene variants in MS onset and disease course in terms of expectation of disability and severity. Although, in our survey the homozygous prevalence of the investigated SNPs is low, ranging from 3% to 8%, we have to take into account that more than 80% of our patients carry at least one of these variants, and that about 50% are double carrier. On the basis that, combined carriers can have phenotypic effects greater than or comparable to single homozygotes, and that iron homeostasis is multi-genetically tuned, this opens new clinical concrete perspectives in monitoring iron accumulation as an underlying mechanism connected to the natural history of MS together with the prognostic value of iron trafficking genes. People carrying at risk alleles could be selected in advance for therapeutic trials aimed to iron chelation and dietary modification in the view that MS course could be in part genetically targeted. So, further larger investigations on iron genes should become mandatory in MS. Understanding the exact mechanism by which iron acts in the brain causing MS and how the brain would be impacted by iron chelation/supplementation could potentially furnish precious prognostic information and novel insights for alternative personalized treatments (pharmacogenetics) aimed in preventing or counteracting neuron loss and degeneration.

All this is in line with a recently published review, on the importance of individualised therapy in MS, based on genetic and biochemical determinations [59].

## Competing interests

The authors declare that they have no competing interests.

## Authors' contributions

DG and PZ were responsible for the core design and content of the report and had access to all aspects of the data. PZ, FS, IB, SDA, MAL, were responsible for enrolment of participants at their sites, furnished clinical patient details and clinically revised the manuscript. GZ, EO, FEDG, CDO, and AVS were responsible for molecular biology techniques and SNPs analyses. DG and RA performed statistical analyses. DG and PZ recruited funds and wrote the paper. All authors have reviewed and approved the content of the manuscript.

## Pre-publication history

The pre-publication history for this paper can be accessed here:

http://www.biomedcentral.com/1471-2350/13/70/prepub
